# Editorial: Quantitative Systems Biology for Engineering Organisms and Pathways

**DOI:** 10.3389/fbioe.2016.00022

**Published:** 2016-03-08

**Authors:** Hilal Taymaz-Nikerel, Alvaro R. Lara

**Affiliations:** ^1^Department of Chemical Engineering, Bogazici University, Istanbul, Turkey; ^2^Departamento de Procesos y Tecnología, Universidad Autónoma Metropolitana-Cuajimalpa, Mexico City, Mexico

**Keywords:** metabolic engineering, systems biology, industrial biotechnology, -omics, regulation

**The Editorial on the Research Topic**

**Quantitative Systems Biology for Engineering Organisms and Pathways**

The biological production of chemicals has gained interest due to its contribution to greener and sustainable processes. Discovering the metabolic capacities of microorganisms shows ascending promise. The understanding of the metabolism and its complex interactions within the process environment is crucial to successfully apply and design cell factories. With the advances of high throughput measurements of -omic levels in a cell, it is possible to decipher the knowledge at different biological processes as a whole. Combining the gained experimental data with computational methods, namely, employing systems biology, allows the development of new bio-products and new cell factories.

This Research Topic of *Frontiers in Bioengineering and Biotechnology* includes six reviews, two mini-reviews, an opinion, and an original research article. Mainly, the importance of applying systems biology approaches for finding metabolic engineering targets is discussed. Valgepea et al. explained their opinion on the potential of proteome optimization in contribution to feasible development of bioprocesses. Delvigne et al. summarized the importance of using fluorescent reporter libraries for the optimization of microbial production under bioreactor environments. They focused on the current status of this technique in terms of methods and applications. Martínez et al. reported the rational design examples of *Escherichia coli* strains for the production of shikimic acid, a precursor aromatic compound in the synthesis of a drug, which is efficient against diverse viruses, including H5N1 and H1N1. In addition, they discussed the challenging tasks required for further improving the overproducing strains using global transcriptomic analyses. Vargas-Tah and Gosset explored the microbial production of two other aromatic intermediates: cinnamic and *p*-hydroxycinnamic acids. The approaches in metabolic engineering of various microorganisms to optimize the usage of raw material and increase the efficiencies of these products were explained. Licona-Cassani et al. provided recent efforts on the systems biology studies in actinomycetes, which are important pathogens and valuable sources of antibiotics, in relation to optimize the production of bioactive natural products. Ates evaluated another example on the application of omics technologies in her review, the production of microbial exopolysaccharides. In addition to these studies on the application of systems biology tools for industrial-scale production, Freudenau et al. presented a dynamic mathematical model constructed to understand the factors affecting the production of plasmid DNA as a pharmaceutical gene vector.

Blombach and Takors reviewed the impacts of carbon dioxide/bicarbonate levels on the physiological, production, metabolism, and regulation processes in microbial and mammalian cultures, a relevant issue still poorly understood. Caspeta et al. focused on the lignocellulosic production of ethanol in *Saccharomyces cerevisiae* and reviewed the information available on the inhibitory conditions during such processes and related stress response mechanisms of the cells. Understanding the reprograming of cellular functions in response to stress environments is crucial, and Taymaz-Nikerel et al. reviewed the current knowledge gained mainly by transcriptomic studies carried out in *S. cerevisiae*. Furthermore, they addressed the requirements of construction of a quantitative whole cell model, which is the ultimate goal of systems biology.

We expect that the developments in this field will continue to increase, eventually yielding quantitative predictive models, which will be useful in many areas.

## Author Contributions

Both authors participated equally in the preparation of this contribution, have read, and approved the final manuscript.

## Conflict of Interest Statement

The authors declare that the research was conducted in the absence of any commercial or financial relationships that could be construed as a potential conflict of interest.

